# Direct Oral FXa Inhibitors Binding to Human Serum Albumin: Spectroscopic, Calorimetric, and Computational Studies

**DOI:** 10.3390/ijms24054900

**Published:** 2023-03-03

**Authors:** Nory Mariño-Ocampo, Diego F. Rodríguez, Daniel Guerra Díaz, Daniel Zúñiga-Núñez, Yorley Duarte, Denis Fuentealba, Flavia C. Zacconi

**Affiliations:** 1 Escuela de Química, Facultad de Química y de Farmacia, Pontificia Universidad Católica de Chile, Santiago 7820436, Chile; 2 Center for Bioinformatics and Integrative Biology, Facultad de Ciencias de la Vida, Universidad Andrés Bello, Santiago 8370035, Chile; 3 Institute for Biological and Medical Engineering, Schools of Engineering, Medicine and Biological Sciences, Pontificia Universidad Católica de Chile, Santiago 7820436, Chile; 4 Centro de Investigaciones en Nanotecnología y Materiales Avanzados, CIEN-UC, Pontificia Universidad Católica de Chile, Santiago 7820436, Chile; 5 Center for Nanomedicine, Diagnostic & Drug Development (ND3), Universidad de Talca, Talca 3460000, Chile

**Keywords:** FXa inhibitors, human serum albumin, fluorescence, isothermal titration calorimetry, molecular modeling, direct oral FXa inhibitors, commercially available FXa inhibitors, apixaban, rivaroxaban, edoxaban, betrixaban

## Abstract

Direct FXa inhibitors are an important class of bioactive molecules (rivaroxaban, apixaban, edoxaban, and betrixaban) applied for thromboprophylaxis in diverse cardiovascular pathologies. The interaction of active compounds with human serum albumin (HSA), the most abundant protein in blood plasma, is a key research area and provides crucial information about drugs’ pharmacokinetics and pharmacodynamic properties. This research focuses on the study of the interactions between HSA and four commercially available direct oral FXa inhibitors, applying methodologies including steady-state and time-resolved fluorescence, isothermal titration calorimetry (ITC), and molecular dynamics. The HSA complexation of FXa inhibitors was found to occur via static quenching, and the complex formation in the ground states affects the fluorescence of HSA, with a moderate binding constant of 10^4^ M^−1^. However, the ITC studies reported significantly different binding constants (10^3^ M^−1^) compared with the results obtained through spectrophotometric methods. The suspected binding mode is supported by molecular dynamics simulations, where the predominant interactions were hydrogen bonds and hydrophobic interactions (mainly π–π stacking interactions between the phenyl ring of FXa inhibitors and the indole moiety of Trp214). Finally, the possible implications of the obtained results regarding pathologies such as hypoalbuminemia are briefly discussed.

## 1. Introduction

**Figure 1:**
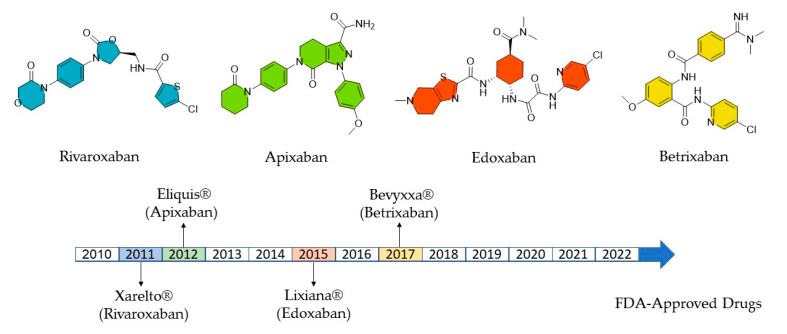
Direct FXa inhibitors approved by the FDA.

Cardiovascular diseases are the leading cause of death worldwide, with 17.9 million reported cases in 2019 alone [1]. Currently, direct FXa inhibitors are among the most widely applied oral anticoagulants for the effective treatment of thromboembolic disorders [2,3]. In this context, Eliquis^®^ (apixaban) and Xarelto^®^ (rivaroxaban), are among the 10 best-selling drugs in the world, with accumulated joint sales of approximately USD 16.1 billion in 2020 alone [4]. These new FXa inhibitors, introduced after 2011 (Figure 1), are effective for the prophylaxis of deep vein thrombosis, strokes, and pulmonary embolism [5,6]. They show various advantages compared with long-established alternatives such as vitamin K antagonists. These advantages include more favorable bleeding profiles, fewer contraindicative drug interactions, a wider therapeutic range, and easier application routes [7,8,9].

A drug’s interaction with plasma proteins is an essential aspect governing its biodistribution and overall response. Generally, the free drug can exert pharmacological activity, while complexes formed with plasma proteins lead to pharmacological inactivation [10]. FXa inhibitors bind reversibly to plasma proteins, forming protein–drug complexes with varying binding affinities, ranging from 55% for edoxaban to 95% for rivaroxaban, respectively [11]. In addition, these differences in affinity have influences on other drug characteristics, such as inertness in response to dialysis procedures [12,13,14], an aspect especially relevant in the context of antithrombotic therapies [15].

Human serum albumin (HSA) is the most abundant protein in the circulatory system (45 g/L, 0.70 mM), comprising 60–65% of the total plasma protein content [16,17]. Its importance is reflected through multiple physiological functions: (i) modulation of the colloid osmotic pressure; (ii) efficient free radical scavenging; (iii) anticoagulant effects; (iv) display of (pseudo-) enzymatic properties, and (v) transport of exogenous and endogenous substances through the system [18,19,20].

**Figure 2:**
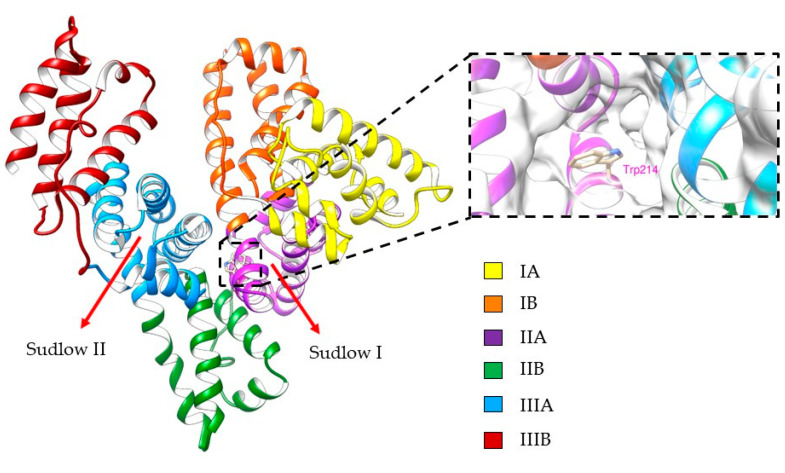
The 3D structure of human serum albumin (HSA) and the localization of its binding sites (PDB: 1AO6).

HSA is a single chain polypeptide composed of 585 amino acids, with a structure consisting of three homologous domains, each of those composed of two sub-domains A and B [21]. It presents two main binding sites suited for the binding of drugs or bioactive compounds, named Sudlow I and Sudlow II (Figure 2) [22]. The elucidation of the binding interactions of drugs to HSA is of utmost importance for the study and prediction of pharmacokinetic and pharmacodynamic properties [23,24,25]. In addition, HSA levels are often used as an indicator for different cardiovascular pathologies [26]. Most recently, a significant relationship between risk of bleeding and levels of HSA in patients receiving rivaroxaban has been reported [27].

Spectroscopic studies of the HSA–apixaban complex were reported by Wang et al. in 2015. In 2020, the interaction of HSA with different anticoagulants was described, analyzing the complexation via absorbance, and the competitive inhibition of different HSA binding sites with fluorescence probes was reported by De Simone et al. [28,29]. Binding of rivaroxaban with bovine serum albumin was subsequently studied by Wani et al. [30]. However, in these reports the association constants were evaluated through spectroscopic and computational methods only, without the consideration of thermodynamic information (e.g., through ITC). Furthermore, there have been no reports about complexes of HSA with more structurally varied anticoagulants [31]. For this reason, more complete research into the interaction of FXa inhibitors and HSA has been lacking. In the current paper, we report an integral approach studying the binding of HSA to FXa inhibitors, using: (i) time-resolved steady-state fluorescence quenching of the excited states of the complexes in Sudlow I, and (ii) isothermal titration calorimetry analyses for the complete thermodynamic study of interactions, enthalpy, entropy, and binding constants of the complexes in the ground state. Considering that there has been no prior complete information detailing these parameters, this research represents the first complete analysis of these novel FXa inhibitors. Additionally, the binding mode and stability were studied using molecular docking and molecular dynamics simulations. The reported results deepen our understanding of the binding modes and affinities of FXa inhibitors towards HSA, providing valuable information relevant for different research areas ranging from drug delivery to cardiovascular medicine.

## 2. Results and Discussion

### 2.1. Fluorescence Quenching of HSA

**Figure 3:**
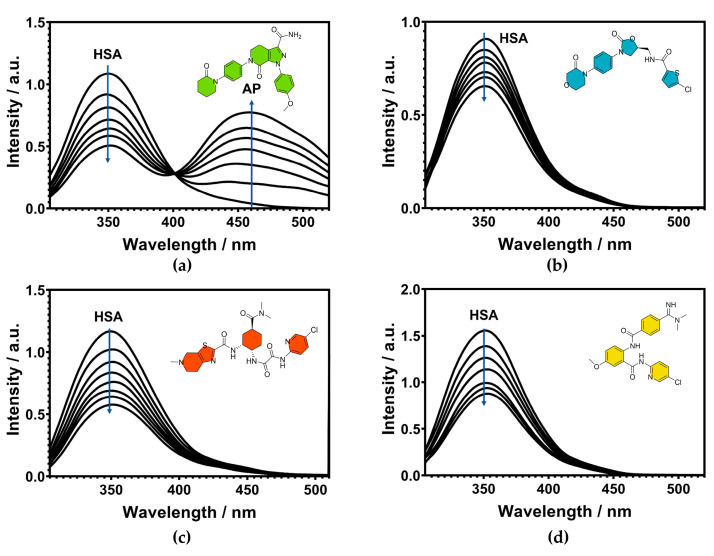
Fluorescence emission spectra of HSA at 298 K in the presence of (**a**) apixaban, (**b**) rivaroxaban, (**c**) edoxaban, and (**d**) betrixaban. Sample measurements were recorded in 0.10 M tris-HCl buffer pH 7.4 with 0.10 M NaCl, 2 µM HSA, 0–20 µM FXa inhibitors respectively (λ_ex_ = 295 nm).

The fluorescence intrinsic to proteins is attributed to aromatic amino acid residues, such as tryptophan (Trp), phenylalanine (Phe), and tyrosine (Tyr) [32]. The fluorescence of HSA in particular can be traced back to its tyrosine and tryptophan residues, therefore the experiments were performed with an excitation wavelength of 295 nm, because tryptophan absorbs light in this range [33]. HSA has a single tryptophan residue (Trp-214) close to the Sudlow site I (subdomain IIA), responsible for the main fluorescence emission of the protein [34,35]. Binding to this site induces fluorescence quenching, which is a very helpful tool to evaluate different drugs’ interactions with HSA. In the present study, the HSA concentration was kept constant at 2 µM, and increasing concentrations of anticoagulants or FXa inhibitors such as apixaban, rivaroxaban, edoxaban, betrixaban and their respective salts were utilized (0–20 µM) [36]. The fluorescence emission spectra of HSA showed a decrease in intensity of 28–51%, indicating efficient fluorescence quenching of the Trp-214 residue (Figure 3 and Figures S1 and S2, Supplementary Information).

HSA exhibits an emission maximum at 350 nm [37,38,39] and does not show any appreciable shift during complex formation with anticoagulant drugs. Furthermore, the anticoagulant drugs showed no notable emissions, except for apixaban, which showed a strong peak at 460 nm (Figure 3a), in line with values previously reported in the literature [28]. Nevertheless, the binding constant of the HSA-AP complex was determined by the HSA quenching analysis, not by the change in the AP fluorescence, as commonly used with the other drugs.

**Figure 4:**
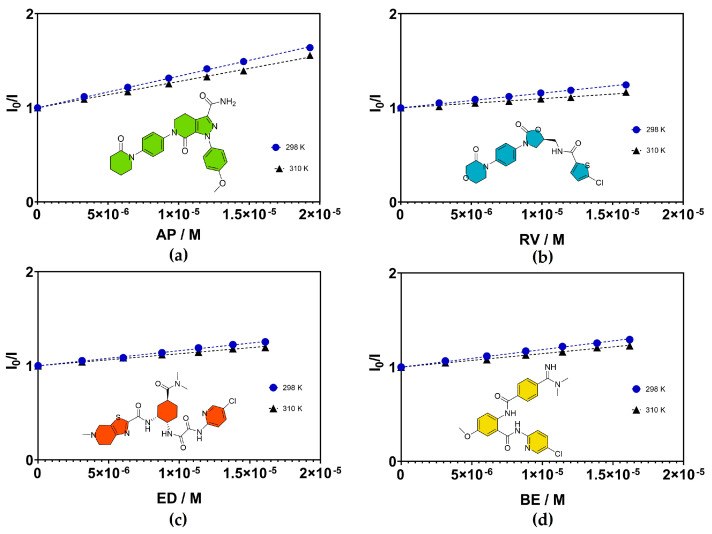
Stern–Volmer quenching plots for HSA with: (**a**) apixaban, (**b**) rivaroxaban, (**c**) edoxaban, and (**d**) betrixaban, at 298 K and 310 K.

The quenching process of HSA can be described as static or dynamic. Static quenching occurs when HSA forms complexes with anticoagulants in the ground state, whereas dynamic quenching occurs via diffusion-controlled encounters in the excited state [40]. Consequently, temperature affects the quenching processes in a different manner. The Stern–Volmer constant has a temperature dependence: K_sv_ in a dynamic process increases with temperature due to the necessity of successful diffusion and collision of the FXa inhibitors with the tryptophan residues of HSA. In a static quenching process, K_sv_ decreases with rising temperature, due to the diminished stability of the complex in the ground state [32,41]. The quenching process of HSA can be analyzed by the Stern–Volmer equation, which provides helpful information regarding the binding process. Table 1 and Figure 4 summarize the K_sv_ of the HSA-FXa inhibitor complexes at 298 K and 310 K respectively. The K_sv_ of betrixaban and edoxaban were compared with their respective salts, betrixaban maleate (BEM) and edoxaban tosylate hydrate (EDT). However, they showed no significant differences compared to the neutral compounds (Figures S1–S3 and Table S1, see Supplementary Information).

The Stern–Volmer constants in Table 1 show a tendency to decrease with increasing temperature, suggesting a static quenching mechanism. Static quenching occurs when HSA forms complexes with the anticoagulants in the ground state, while dynamic quenching takes place via diffusion-controlled encounters in the excited state [37]. However, this trend by itself is insufficient to prove that static quenching is taking place, since the studies were performed at only two temperatures. Therefore, we determined fluorescence-lifetime measurements [32]. The fluorescence lifetimes did not vary for complexations in the ground state, which is when static quenching occurs; however, for the dynamic interactions with the excited state, decreases in the decay time for the entire excited-state population were observed [28]. The fluorescence lifetimes of complexes between HSA and anticoagulants (molar relation of 1:1) are summarized in Table 2. Analyses of the fluorescence lifetime of complexes with different molar ratios can be found in Tables S2–S5 (see Supplementary Information). The molar relations between has and the FXa inhibitors were 1:1, 1:2, 1:4, and 1:6. For all cases, the fluorescence lifetimes did not change appreciably, confirming the occurrence of complexation between HSA and FXa inhibitors in the ground state, thus inducing static quenching of the Trp fluorescence. The results were analyzed for these two specific temperatures, considering room temperature and physiological temperature for the drugs of interest. It is important to remark that in such a scenario, the K_sv_ is equivalent to the binding constants for the HSA-anticoagulant complexes, in the range of (1–3) × 10^4^ M^−1^. Considering that HSA binding data were available only for apixaban, this research study represents the first complete fluorescence quenching analyses of HSA for the four described FXa inhibitors.

### 2.2. Thermodynamic Studies by Isothermal Titration Calorimetry

ITC analyses were performed in phosphate buffer, 10 mM, pH = 7.4. The phosphate buffer was preferred over tris-HCl buffer used in the fluorescence titrations, since it exhibits lower ionization enthalpy (pK = 7.198; Δ_f_H° = 0.86 kcal mol^−1^) than tris-HCl (pK = 8.072; Δ_f_H° = 11.34 kcal mol^−1^) [42]. Lower ionization enthalpy reduces heat signals associated with proton-exchange reactions between the buffer and the ligand/host system during complex formation. ITC studies were attempted for all reported drugs, including the salts of BE and ED, i.e., BEM and EDT, due to their greater water solubility. However, only AP and BEM showed heat-evolution signals associated with the binding processes, compared with the control dilution experiments (drug over buffer). RV, BE, ED, and EDT demonstrated poor aqueous solubility even with solvent mixtures that included ethanol and DMSO [43]. Figures S4 and S5 (Supplementary Information) show the ITC data for the titration of AP and BEM in phosphate buffer, 10 mM, pH = 7.4, at 298 K (top of the image). A plot of the heat released per mole of HSA as a function of the molar ratio of the solution ([HSA]/[anticoagulant]) is shown at the foot of each figure. A one-site binding model showed the best fit with the data, and is therefore included in each figure. Control titrations were performed (drug over buffer) and subtracted from each analysis before the fitting. The binding constants and thermodynamic parameters are shown in Table 3. AP showed a higher binding constant compared with BEM (Table 3), similar to the observations for the fluorescence-quenching experiments (Table 1). The difference in magnitude of the binding constants obtained through ITC or fluorescence is due to the fact that the binding process was measured in the ground state or excited state, respectively [42]. Different heat-evolution behaviors (endothermic or exothermic) were due to the nature of the anticoagulant used, and the way the titration was performed. This is the first complete calorimetric study to include all the current commercial oral direct inhibitors of FXa binding to fatty-acid-free HSA.

The binding affinity of HSA and BEM is governed by different interactions between the anticoagulants and the HSA protein in the aqueous medium in which they are dissolved. These interactions, the rotational degrees of freedom of the inhibitors, and the conformational changes in the protein contribute directly to the protein–ligand binding energy [44]. From the thermodynamic perspective, binding affinity is determined by the magnitude of the Gibbs energy (ΔG), which is composed of enthalpy changes (ΔH) and entropy changes (ΔS), both of which contribute to the binding energy (ΔG = ΔH − TΔS). The enthalpy changes upon binding are due to the strength of the interactions between the anticoagulants/HSA relative to those of the solvent. The favorable contribution to enthalpy arises mainly from van der Waals interactions, electrostatic interactions, and hydrogen bonds between the anticoagulants and the protein. The net enthalpy change is a result of the combination of these contributions. On the other hand, entropy is associated with the interaction of the hydrophobic groups with the solvent, and the loss of conformational degrees of freedom involved in the binding [45].

The variations between thermodynamic parameters are evidence of an enthalpy–entropy trade-off [46]. According to the ITC results, as ΔH becomes more negative, ΔS tends to decrease due to multiple favorable non-covalent interactions between the anticoagulant/HSA complex. Conversely, as ΔH becomes less negative, ΔS tends to increase as the complex becomes increasingly disordered.

The ITC measurements indicate that the binding of BEM to HSA is driven by a favorable negative change in enthalpy (ΔH = −3.22 kcal mol^−1^), whereas in AP/HSA binding, the enthalpy change is unfavorable (ΔH = 6.21 kcal mol^−1^). However, the conformational entropy values compensate for this energy loss, thus achieving negative free energy binding for the two complexes (lower than −4.0 kcal mol^−1^). Docking studies (see below) show a higher coulombic contribution for the binding of BE compared with AP, which could be associated with the imine group electric charge in BE. BEM has a positive charge in the imine group, increasing the coulombic contribution to the binding, leading to stronger interaction with Asp451 residue and a more negative enthalpy value.

### 2.3. Molecular Dynamics Simulations

**Figure 5:**
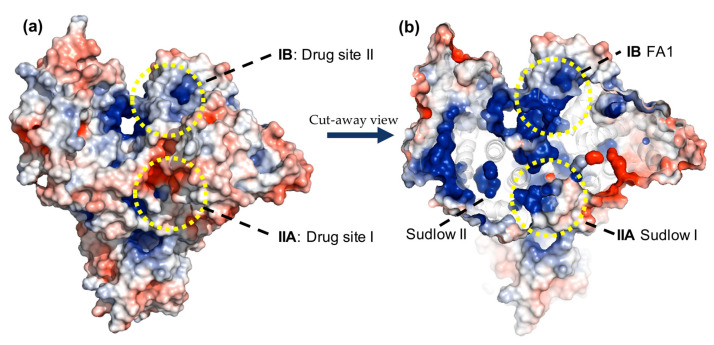
(**a**) Overhead view of the HSA protein in surface representation, colored according to the electrostatic surface potential. (**b**) Cutaway view of the HSA protein shows internal multiple binding sites with mainly positive electrostatic potential.

Crystallographic analysis showed that HSA contains multiple binding sites with positive residues to which several molecules can bind through hydrogen bonding, salt bridges, and π–cation interactions (Figure 5). Two primary sites, Sudlow I and Sudlow II in subdomains IIA and IIIA, respectively, are highly adaptable and flexible for the binding of bioactive compounds [47]. However, the site in subdomain IB has shown favorable binding to some FXa-inhibitors [29]. Aiming to explain the differences in activity of FXa inhibitors and elucidating how these compounds interact with HSA at the molecular level, we explored their behavior using molecular modeling methods. The intermolecular interaction studies and free energy calculations (MM/GBSA) from molecular dynamic (MD) simulations support discussion of the interactions of FXa inhibitors with HSA in the binding sites of the protein: Drug sites I and II (Figure 5). Although the experimental analysis was performed only for site I, we also performed an MD simulation for site II, also called the FA1 site, to compare the ligand affinities of these two possible binding sites [29]. We used crystal structures of ligand-free HSA (PDB ID: 1AO6) [48] to perform our simulations. The three-dimensional structures of apixaban, rivaroxaban, edoxaban, and betrixaban were prepared using ChemDraw and optimized with ligPrep in the Schrodinger suite [49], performing molecular docking studies using the glide software package to obtain initial poses for all FXa inhibitors [50].

Drug site I is a binding pocket located within the core of subdomain IIA, predominantly apolar, bearing a central hydrophobic zone delineated by Phe211 and Trp214 residues, but containing positive residues (Lys195, Lys199, Arg218, Arg222) at the pocket entrance. Drug site II is a binding pocket located in domain IB, with a polar hydrophobic zone, delineated by Tyr161 and Tyr138 residues, and with positively charged residues (Arg186 and Arg117) surrounding the binding pocket, as evidenced by the electrostatic potential on its surface. We performed molecular simulations of 200 ns for each of the four inhibitors at both drug sites, focusing on the most characteristic ligand–receptor interactions presented in drug site I.

**Figure 6:**
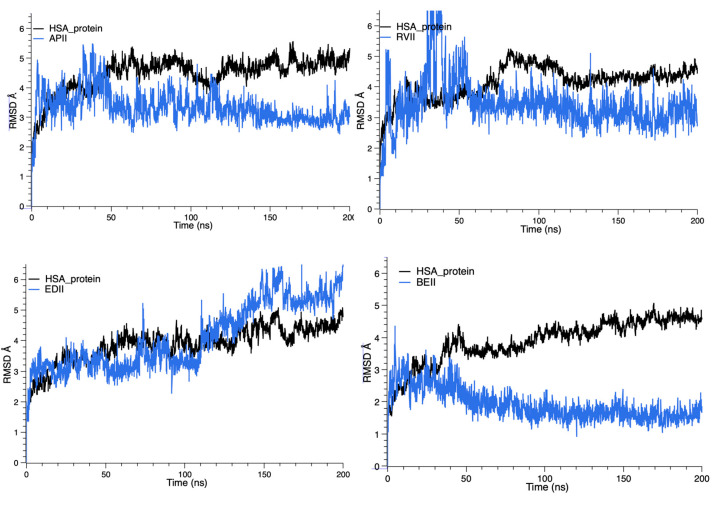
RMSD evolution for four HSA-FXa inhibitor complexes.

We evaluated the stability of MD simulations through RMSD of the backbone atoms in HSA, based on MD trajectories. Figure 6 shows RMSD for the HSA-FXa inhibitor complexes across the simulation time, for drug site I. It can be observed in the RMSD graphs that FXa inhibitors presented instability during the first 100 ns of the simulation but achieved stability at the binding site after this time, except for the case of ED, which showed high fluctuation over the full simulation. The RMSD for HSA-FXa inhibitor complexes in drug site II indicated similar behavior to site I, but with higher stability during all the trajectories using ED as the inhibitor, and with higher levels of fluctuation.

**Figure 7:**
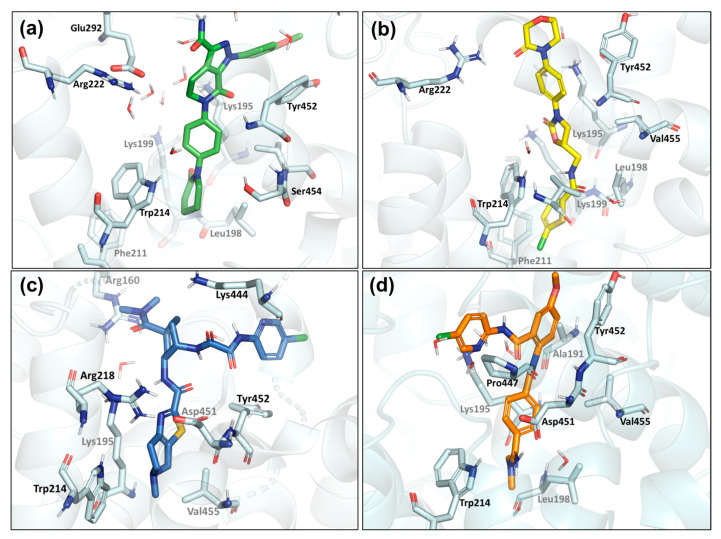
Representative binding mode of the FXa inhibitors at the HSA binding site 1: (**a**) apixaban in green, (**b**) rivaroxaban in yellow, (**c**) edoxaban in blue, and (**d**) betrixaban in orange.

In the MD simulations, the different FXa inhibitors occupied the drug site I in HSA to similar extents, revealing only small side-chain movements associated with size, adaptability, and drug conformation on the binding site. Three compounds, AP, RV, and ED, showed hydrophobic interactions upon access to the binding pocket’s central zone, mainly π–π stacking interaction between the π electrons of the phenyl ring and the indole ring of Trp214, with distances of less than 5.0 Å, whereas only AP and RV notably interacted with Phe211 (Figure 7). In addition to hydrophobic contact, the residue Lys195 plays a protagonist role in several interactions, including hydrophobic hydrogen bonding, and water-mediated hydrogen bonding interactions with carbonyl oxygen atoms in all compounds. Equally, interactions involving the π-electron systems, such as the phenyl moieties of FXa inhibitors or other aromatic residues, can include cation and π–cation interactions with positively charged molecules or amino acids. Therefore, Trp214 and Lys195 assume central roles in HSA-FXa inhibitor interactions. Strikingly, although BE did not show interactions with Trp214 and Lys195 residues, it showed high stability during the simulation, due to strong hydrogen-bonding contact between the imine moiety of BE and Asp451, and hydrophobic interaction of the phenyl group with the Tyr452 residue, respectively (Figure 7d). The interactions of FXa inhibitors in site II of the HSA featured mainly π–π stacking and hydrogen bond interactions between the respective FXa inhibitors and Tyr138 and Tyr161 residues, with distances of less than 3.0 Å. In addition, the FXa inhibitors are stabilized through hydrogen bonding, and cationic and π–cation contact with Arg114, Arg145, and Arg186 residues.

In the simulation, we evaluated the binding energy for each HSA-FXa inhibitor complex, using MM/GBSA (molecular mechanics generalized born surface area) analysis. We calculated the energy components of each system from the last 100 ns of the production trajectories (Table 4). Among the evaluated FXa inhibitors in drug site I, BE and AP have the most favorable free energy values ΔG_GB_ (−73.23 ± 4.37 and −72.36 ± 4.43 kcal mol^−1^, respectively). However, a notable difference exists in the energetic contributions of these two drugs, mainly regarding their Coulomb energy (ΔE_coul_). The Coulomb contributions for AP and BE were −22.47 ± 4.38 and −51.79 ± 12.41, respectively. The high share of coulombic or electrostatic contribution could be associated with the strong electric charge of the imine group present in BE and its close interaction with the Asp451 residue in HSA. For AP, the most significant contribution was van der Waals energy ΔE_VdW_. These values were lower than other energetic terms, suggesting that the hydrophobic interaction was a major contributor to the overall ligand binding. The free energy values in drug site II presented similar behavior to drug site I, however, exhibiting more negative values.

The ΔG_GB_ values for both AP/HSA and BEM/HSA showed a thermodynamic compensation in relation to the energy terms involved. Although we cannot directly compare the theoretical energy values with the thermodynamic parameters obtained via ITC, due to the difficulty in sampling the bound and free states of the binding complexes from MD simulations, we can relate the intermolecular interactions relating to enthalpy. The binding enthalpy is the result of energetic changes obtained from the combination of non-covalent interactions in the binding complex (van der Waals contacts, electrostatic interactions, hydrogen bonds, and any other polar or non-polar interactions). In this sense, the ΔE_VdW_ and ΔE_coul_ contributions were the most representative in terms of the binding-energy value and, therefore, those that contributed the most to the described enthalpy.

### 2.4. Possible Clinical Implications of the Binding of Direct FXa Inhibitors to HSA

Warfarin is a vitamin K antagonist (VKA) with significant therapeutic drawbacks such as a narrow therapeutic window, serious side effects including skin necrosis and danger of fetal abnormalities in pregnant women, detrimental drug–drug and drug–food interactions, high risk of bleeding, unfavorable distribution, slow renal clearance, and the need for frequent monitoring [51,52,53]. Warfarin affects the synthesis of blood proteins (FII, FVII, FIX, FX) which are vitamin K-dependent clotting proteins [54]. Additionally, hypoalbuminemia is related to an increased risk of bleeding in patients treated with warfarin. Since this VKA binds mainly to albumin, in patients with this pathology the free fraction of warfarin is higher, which increases its anticoagulant effects, inducing enhanced bleeding [55].

On the other hand, direct oral FXa inhibitors (rivaroxaban, apixaban, edoxaban, and betrixaban) are specific inhibitors for this blood protein and have been shown to be selective in comparison with warfarin [56,57,58]. Moreover, these inhibitors present only very few drug–drug and drug–food interactions, do not require strict dose monitoring protocols, have higher volumes of distribution (21–107 L), and are partly renally cleared [59,60,61,62].

The level of HSA is an important prognostic factor for different pathologies [63,64]. In addition, hypoalbuminemia (HSA < 3.5 g/dL) is a strong independent predictor for diverse cardiovascular pathologies such as venous thromboembolism (VTE) and stroke [26,65]. Under these conditions, it has been reported that the pharmacokinetics of several drugs may be affected by interactions with HSA, making dose adjustments necessary [66]. In fact, low plasma serum albumin levels have been linked to an increased risk of bleeding when using warfarin, a commonly used oral anticoagulant. The enhanced anticoagulant effect of warfarin under physiological conditions has been associated with a higher fraction of the free drug in the blood, and a high binding constant to HSA (from 4 × 10^4^ to 7 × 10^4^) [22,67]. According to the binding constants reported in this work, the direct FXa inhibitors apixaban, rivaroxaban, edoxaban, and betrixaban can exhibit similar behaviors to warfarin in patients with hypoalbuminemia. Actually, a study by Wojakowski et al. in 2020 found that albumin levels are associated with bleeding risk in rivaroxaban-treated patients, whereby each 1.0 g/dL incremental decrease in albumin resulted in a 4.4-fold higher adjusted risk of bleeding [27]. Furthermore, FXa inhibitors showed effectiveness against VTE, associated with advanced gastric cancer, and apixaban is specifically recommended for patients with severe hypoalbuminemia to prevent thrombosis in severe nephrotic syndrome [68].

Previous work reported in the literature used fluorescence or absorption spectroscopies to assess the binding interactions of apixaban with HSA, while for other drugs the data were obtained using either the bovine homologue of the protein (BSA), or in the presence of heme-Fe(III) (Table 5). Therefore, this is the first study to report thermodynamic binding data measured via ITC for two of these commercially available drugs. Because ITC requires the use of much higher concentrations compared with spectroscopic measurements, the low water solubility of rivaroxaban and edoxaban did not allow the generation of ITC data, even in the presence of co-solvents. On the other hand, this is the first complete study of molecular dynamic simulations linked to MM/GBSA analyses for any of the four commercially available FXa inhibitors, and, according to these results, either site may be suitable for their binding to HSA.

These facts emphasize the importance of a comprehensive evaluation for new approved drugs of their association constants with HSA, if possible, using a comprehensive approach and avoiding overestimation of the results.

## 3. Materials and Methods

### 3.1. Preparation of Stock Solutions

Fatty-acid-free human serum albumin (HSA) was purchased from Sigma-Aldrich and used without purification. Stock solutions of HSA were prepared in tris-HCl buffer at pH 7.4 with 0.10 M NaCl. Apixaban (AP), rivaroxaban (RV), and betrixaban (BE) were purchased from Ambeed Inc. Edoxaban (ED). Edoxaban tosylate hydrate (EDT) and betrixaban maleate (BEM) were purchased from AKScientific. Stock solutions of AP, RV, ED, BE, EDT, and BEM were prepared in DMSO. For the complexation studies using HSA and the anticoagulants, the added DMSO was less than 2% [28].

### 3.2. Materials and Equipment

#### 3.2.1. Fluorescence Measurements

Fluorescence emission spectra were collected on a Perkin Elmer LS55 fluorimeter, using 5 nm bandwidths for excitation and emission. Samples were excited at 295 nm and 298 K or 310 K, respectively. Subsequently, HSA fluorescence was measured at 350 nm decreases, in the presence of different anticoagulants, corrected by the inner-filter effect using Equation (1) where *I_corr_* and *I_obs_* correspond to fluorescence corrected and obtained intensities, and *A_ex_* and *A_em_* correspond to the absorbance at the excitation and emission wavelengths [32,41,69]. The quenching data were evaluated using the Stern–Volmer Equation (2), where *I_0_* and *I* are the fluorescence intensities of the HSA in the absence or presence of anticoagulants (Q, quenchers), and *K_sv_* is the Stern–Volmer constant:

(1) $$I_{corr}=I_{obs}\times 10^{\left(\frac{A_{ex}+A_{em}}{2}\right)}$$

(2) $$\frac{I_{0}}{I}=1+K_{sv}\left[Q\right]$$

Fluorescence lifetimes were determined using the TCSPC technique in a LifeSpecII fluorescence spectrometer (Edinburgh Instruments). The samples were excited using a light-emitting diode (LED) of 279 nm, which was the closest available to the HSA absorption maximum, in tris-HCl buffer at pH 7.4 with 0.1 M NaCl. The emission data were collected to 5000 counts. The instrument response (IRF) was evaluated by scattering the excitation light, using a diluted Ludox solution. The fluorescence decays were fitted using Equation (3), where the pre-exponential factor *A_i_* is related to the contribution of one species to the intensity of the decay, and τ_1_ corresponds to the fluorescence lifetime of a different species. The goodness of fit was judged according to the residual distribution around zero and $\chi^{2}$-values between 0.9 and 1.2 [70]. All the measurements were performed in triplicate.

(3) $$I\left(t\right)=I_{0}\sum_{1}^{i}A_{i}e^{-t/\tau_{i}}$$

#### 3.2.2. Isothermal Titration Calorimetry (ITC)

ITC data were obtained using a MicroCal PEAQ-ITC instrument (Malvern Panalytical). Stock solutions of anticoagulants were prepared in DMSO (1.58–8.27 mM), except for betrixaban maleate, for which the stock solution was prepared directly in phosphate buffer, 10 mM, pH = 7.4. Working solutions were prepared using aliquots of the stock solutions of anticoagulants diluted in phosphate buffer, 10 mM, pH = 7.4. Final concentrations of anticoagulants in buffer were around 30–50 µM, depending on each compound’s solubility in water. Human serum albumin (HSA) stock solution was prepared in the same buffer (700–800 µM), and the concentration was assessed by UV-Vis spectroscopy, using a molar extinction coefficient of 35,700 M^−1^ cm^−1^ at 280 nm [71]. The first injection was 0.40 μL, followed by 12 injections of 3.0 μL. Reference power was set to 5 μcal/s, 500 rpm stirring speed, initial delay of 60 s, and injection spacing of 150 s. Titration curves were drawn in duplicate and the thermodynamic parameters were fitted to the one-site binding model using Microcal PEAQ-ITC analysis software. Controls were measured by recording the heat effects of the titrant over plain buffer and were subtracted from thermograms before fitting analysis. All the measurements were performed in duplicate.

#### 3.2.3. Computational Analysis

The HSA-FXa inhibitor structures obtained from the molecular docking calculations were prepared and studied through MD simulation, to acquire the HSA-FXa inhibitor energy data and identify the residues involved in the main interactions. MD simulations were performed using Desmond, implemented in the Schrödinger suite [50]. The OPLSe and TIP3P force fields were employed to describe FXa inhibitors, HSA protein, and water, respectively. All HSA-FXa inhibitor complexes were neutralized with 0.15 mol L^−1^ of NaCl. Then, the production MD of 200 ns was run, with the coordinates recorded every 10 ps. Molecular dynamic simulations were performed with a 2 fs time step and employing Langevin dynamics for temperature control. Three-dimensional structures for HSA-FXa inhibitor complexes and MD trajectory analyses were inspected with the maestro-Schrödinger graphical interface and PyMOL software package [72].

Finally, the binding affinity for HSA-FXa inhibitors, expressed as ΔG_GB_, was estimated using MM/GBSA methods, using the prime module from the Schrödinger suite with its default settings [50].

## Figures and Tables

**Table 1:** Stern–Volmer constants (K_sv_) for HSA with anticoagulants at two different temperatures.

Anticoagulant	K_sv_ (×10^4^ M^−1^) 298 K	K_sv_ (×10^4^ M^−1^) 310 K
AP	3.36 ± 0.03	2.79 ± 0.02
RV	1.56 ± 0.03	0.98 ± 0.04
ED	1.60 ± 0.04	1.28 ± 0.05
BE	1.86 ± 0.03	1.44 ± 0.04

**Table 2:** Fluorescence lifetimes and pre-exponential factors of complexes of HSA with AP, RV, ED, and BE (1:1) under aerated conditions at 293 K. The excitation wavelength was 279 nm.

Complex	τ_1_ (ns)	A_1_	τ_2_ (ns)	A_2_	χ^2^	<τ_F_>
AP@HSA	3.22	0.45	7.01	0.55	1.10	5.30
RV@HSA	3.00	0.40	6.80	0.60	1.13	5.28
ED@HSA	2.99	0.39	6.80	0.61	1.13	5.31
BE@HSA	3.03	0.39	6.78	0.61	1.10	5.32

**Table 3:** Binding constants and thermodynamic parameters for inclusion complexes of anticoagulants with HSA in phosphate buffer 10 mM, pH = 7.4 at 298 K, determined by ITC.

Complex	K_ITC_ (M^−1^)	ΔH (kcal mol^−1^)	−TΔS (kcal mol^−1^)	ΔG (kcal mol^−1^)
AP@HSA	5.56 × 10^3^	6.21	−11.33	−5.11
BEM@HSA	1.60 × 10^3^	−3.22	−1.14	−4.37

**Table 4:** The calculated binding energy (kcal mol^−1^) for HSA-FXa inhibitor complexes.

Complex	ΔE_VdW_	ΔE_coul_	ΔE_lipo_	ΔG_GB_^[a]^
AP@HSA	−63.57 ± 3.77	−22.47 ± 4.38	−23.40 ± 1.61	−72.36 ± 4.43
RV@HSA	−60.35 ± 3.49	−10.90 ± 3.16	−23.87 ± 1.32	−69.38 ± 3.59
ED@HSA	−62.42 ± 3.13	−14.99 ± 4.44	−18.87 ± 1.52	−61.07 ± 5.71
BE@HSA	−62.15 ± 2.12	−51.79 ± 12.41	−21.64 ± 0.78	−73.23 ± 4.37

^[a]^ Energies: ΔE_VdW_: van der Waals energy; ΔE_coul_: Coulomb energy; ΔE_lipo_: lipophilic energy; ΔG_GB_: estimated binding free energy.

**Table 5:** Literature data for FXa inhibitor interactions and different serum albumins.

FXa Inhibitor	Protein	n	K (M^−1^)	pH	T (°C)	Buffer (Concentration or Ionic Strength)	Binding Site	Experimental Technique	Refs.
Apixaban	HSA fatty acid-free	1.03	3.41 × 10^3^	7.4	28	Tris–HCl (0.10 M NaCl) DMSO 2%	IIA	FS	[26]
Apixaban	HSA fatty acid-free	1.00	2.46 × 10^3^	7.4	37	Tris–HCl (0.10 M NaCl) DMSO 2%	IB	FS	[26]
Apixaban	HSA fatty acid-free	0.97	1.67 × 10^3^	7.4	45	Tris–HCl (0.10 M NaCl) DMSO 2%	IB	FS	[26]
Apixaban	HSA: heme-Fe(III)		2.78 × 10^6^	7.3	20	Phosphate 2.0 × 10^−2^ M DMSO 10%	IB	UV	[27]
Rivaroxaban	BSA	1.10	1.32 × 10^5^	7.4	25	Phosphate DMSO 0.3%	IIA	FS	[28]
Rivaroxaban	BSA	0.98	1.82 × 10^4^	7.4	30	Phosphate DMSO 0.3%	IIA	FS	[28]
Rivaroxaban	BSA	0.85	4.37 × 10^3^	7.4	35	Phosphate buffer DMSO 0.3%	IIA	FS	[28]
Rivaroxaban	HSA: heme-Fe(III)		4.55 × 10^5^	7.3	20	Phosphate 2.0 × 10^−2^ M DMSO 10%	IB	UV	[27]
Betrixaban	HSA: heme-Fe(III)		1.61 × 10^6^	7.3	20	Phosphate 2.0 × 10^−2^ M DMSO 10%	IB	UV	[27]
Edoxaban	HSA: heme-Fe(III)		7.69 × 10^5^	7.3	20	Phosphate 2.0 × 10^−2^ M DMSO 10%	IB	UV	[27]

Acronyms. BSA: Bovine serum albumin; FS: fluorescence spectroscopy; UV: ultraviolet–visible spectroscopy.

## Data Availability

The data presented in this study are available in the Supplementary Material.

## Supplementary Materials

The following supporting information can be downloaded at: https://www.mdpi.com/article/10.3390/ijms24054900/s1.
