# Supplementary material for: Direct Oral FXa Inhibitors Binding to Human Serum Albumin: Spectroscopic, Calorimetric, and Computational Studies
Source: Int J Mol Sci. 2023 Mar 3;24(5):4900. doi: 10.3390/ijms24054900 (PMC10002493; doi:10.3390/ijms24054900)
Supplement: Supplementary file 1 [file ijms-24-04900-s001.zip › ijms-2165522-supplementary.pdf]

SUPPLEMENTARY MATERIAL

Direct Oral FXa Inhibitors Binding to Human Serum Albumin: Spectroscopic, Calorimetric, and Computational Studies

Nory Mariño-Ocampo [1, co], Diego F. Rodríguez [1, co], Daniel Guerra Díaz [1], Daniel Zúñiga-Núñez [1], Yorley Duarte [2], Denis Fuentealba[1, *] and Flavia C. Zacconi [1,3,4,5*]

1 Escuela de Química, Facultad de Química y de Farmacia, Pontificia Universidad Católica de Chile, Santiago 7820436, Chile.
2 Center for Bioinformatics and Integrative Biology, Facultad de Ciencias de la Vida, Universidad Andrés Bello, Santiago 8370035, Chile.
3 Institute for Biological and Medical Engineering, Schools of Engineering, Medicine and Biological Sciences, Pontificia Universidad Católica de Chile, Santiago 7820436, Chile.
4 Centro de Investigaciones en Nanotecnología y Materiales Avanzados, CIEN-UC, Pontificia Universidad Católica de Chile, Santiago 7820436, Chile.
5 Center for Nanomedicine, Diagnostic & Drug Development (ND3), Universidad de Talca, Talca 3460000, Chile.
[co] Contributed equally to this work: Nory Mariño-Ocampo and Diego F. Rodríguez.
* Correspondence: dlfuente@uc.cl (D.F.), fzacconi@uc.cl (F.C.Z.)

List of content

1. **Figure S1.** Fluorescence quenching spectra of HSA in the presence of edoxaban tosylate at 298 K. Samples measurements were recorded in 0.10 M Tris-HCl buffer pH 7.4 and 0.10 M NaCl, λex=295 nm, and, λem=350 nm……………………………………………………..………………………3S
2. **Figure S2.** Fluorescence quenching spectra of HSA in the presence of betrixaban maleate at 298 K. Samples measurements were recorded in 0.10 M Tris-HCl buffer pH 7.4 and 0.10 M NaCl, λex=295 nm, and, λem=350 nm.…………………………………………………………….……………………3S
3. **Figure S3.** The Stern-Volmer curves for the quenching of HSA by: a) edoxaban tosylate hydrate and b) betrixaban maleate at 298 K and 310 K………..…………………………………..……………………………4S
4. **Table S1.** Stern-Volmer quenching constant (K_{sv}) of HSA with anticoagulants at two temperatures…..………..……………………………4S

5. **Table S2.** Fluorescence lifetimes and pre-exponential factors of HSA (2 μM) with AP at different concentrations (0-12 μM) at 293 K in under aerated conditions. The excitation wavelength was 279 nm…………………..………4S
6. **Table S3.** Fluorescence lifetimes and pre-exponential factors of HSA (2 μM) with RV at different concentrations (0-12 μM) at 293 K in under aerated conditions. The excitation wavelength was 279 nm…………………………5S
7. **Table S4.** Fluorescence lifetimes and pre-exponential factors of HSA (2 μM) with ED at different concentrations (0-12 μM) at 293 K in under aerated conditions. The excitation wavelength was 279 nm……………….………5S
8. **Table S5.** Fluorescence lifetimes and pre-exponential factors of HSA (2 μM) with BE at different concentrations (0-12 μM) at 293 K in under aerated conditions. The excitation wavelength was 279 nm….……………….……6S
9. **Figure S4.** ITC data for a titration of 696 μM HSA into AP solution (50 μM) in phosphate buffer 10 mM, pH = 7.4. *Top:* Raw data; *bottom:* Integrated heat effects per injection as a function of molar ratio (fitted to a one site model)...…………………………………………………………………6S
10. **Figure S5.** ITC data for a titration of 1.58 mM BE maleate into HSA solution (100 μM) in phosphate buffer 10 mM, pH = 7.4. *Top:* Raw data; *bottom:* Integrated heat effects per injection as a function of molar ratio (fitted to a one site model)...…………………………………………………..…..7S

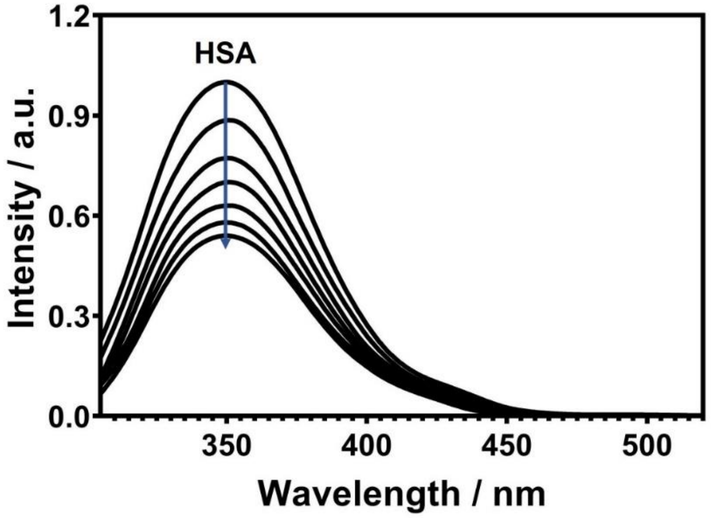

Figure S1. Fluorescence quenching spectra of HSA in the presence of edoxaban tosylate at 298 K. Samples measurements were recorded in 0.10 M Tris-HCl buffer pH 7.4 and 0.10 M NaCl, λex=295 nm, and λem=350 nm.

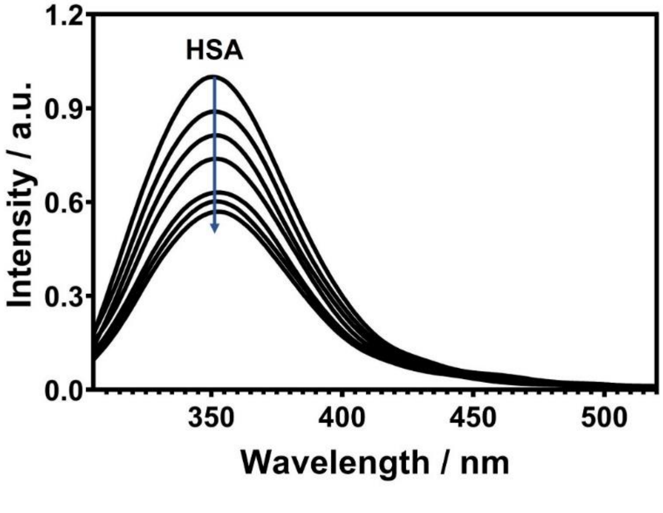

Figure S2. Fluorescence quenching spectra of HSA in the presence of betrixaban maleate at 298 K. Samples measurements were recorded in 0.10 M Tris-HCl buffer pH 7.4 and 0.10 M NaCl, λex=295 nm, and , λem=350 nm.

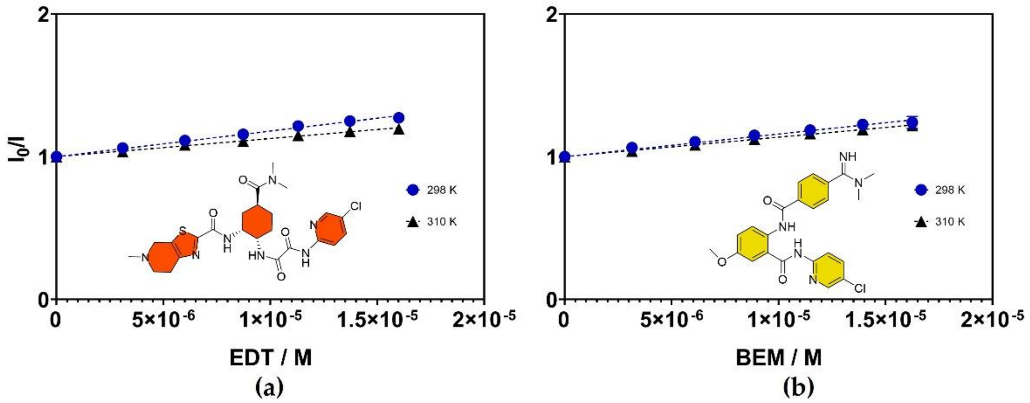

Figure S3. The Stern-Volmer curves for the quenching of HSA by: a) edoxaban tosylate hydrate and b) betrixaban maleate at 298 K and 310 K.

Table S1. Stern-Volmer quenching constant (K_{sv}) of HSA with anticoagulants at two temperatures.

Anticoagulant	K_{sv} ($\times 10^4$ M^{-1}) 298 K	K_{sv} ($\times 10^4$ M^{-1}) 310 K
EDT	1.79±0.03	1.27±0.02
BEM	1.59±0.05	1.36±0.03

Table S2. Fluorescence lifetimes and pre-exponential factors of HSA (2 μM) with AP at different concentrations (0-12 μM) at 293 K in under aireated conditions. The excitation wavelength was 279 nm.

Probe	τ_1 (ns)	A_1	τ_2 (ns)	A_2	χ^2	$<\tau_F>$
HSA	3.01	0.41	6.80	0.59	1.21	5.25
AP@HSA (1:1)	3.22	0.45	7.01	0.55	1.10	5.30
AP@HSA (1:2)	2.93	0.41	6.83	0.59	1.06	5.23
AP@HSA (1:4)	2.73	0.38	6.69	0.62	1.17	5.19
AP@HSA (1:6)	2.86	0.42	6.83	0.58	1.13	5.16

Table S3. Fluorescence lifetimes and pre-exponential factors of HSA (2 μM) with RV at different concentrations (0-12 μM) at 293 K in under aireated conditions. The excitation wavelength was 279 nm.

**Probe**	**τ_1 (ns)**	**A_1**	**τ_2 (ns)**	**A_2**	**χ^2**	**$\langle\tau_F\rangle$**
HSA	3.01	0.41	6.80	0.59	1.21	5.25
RV@HSA (1:1)	3.00	0.40	6.80	0.60	1.13	5.28
RV@HSA (1:2)	2.98	0.41	6.82	0.59	1.09	5.25
RV@HSA (1:4)	2.98	0.42	6.85	0.58	0.99	5.22
RV@HSA (1:6)	2.94	0.43	6.87	0.57	1.12	5.18

Table S4. Fluorescence lifetimes and pre-exponential factors of HSA (2 μM) with ED at different concentrations (0-12 μM) at 293 K in under aireated conditions. The excitation wavelength was 279 nm.

**Complex**	**τ_1 (ns)**	**A_1**	**τ_2 (ns)**	**A_2**	**χ^2**	**$\langle\tau_F\rangle$**
HSA	3.01	0.41	6.80	0.59	1.21	5.25
ED@HSA (1:1)	2.99	0.39	6.80	0.61	1.13	5.31
ED@HSA (1:2)	2.93	0.40	6.80	0.60	1.16	5.25
ED@HSA (1:4)	2.71	0.40	6.71	0.60	1.16	5.11
ED@HSA (1:6)	2.94	0.44	6.88	0.54	1.20	5.01

Table S5. Fluorescence lifetimes and pre-exponential factors of HSA (2 μM) with BE at different concentrations (0-12 μM) at 293 K in under aireated conditions. The excitation wavelength was 279 nm.

Complex	τ_1 (ns)	A_1	τ_2 (ns)	A_2	χ^2	$<\tau_F>$
HSA	3.01	0.41	6.80	0.59	1.21	5.25
BE@HSA (1:1)	3.03	0.39	6.78	0.61	1.10	5.32
BE@HSA (1:2)	2.89	0.38	6.78	0.62	1.10	5.30
BE@HSA (1:4)	2.86	0.38	6.73	0.62	1.09	5.26
BE@HSA (1:6)	2.60	0.35	6.61	0.65	1.09	5.21

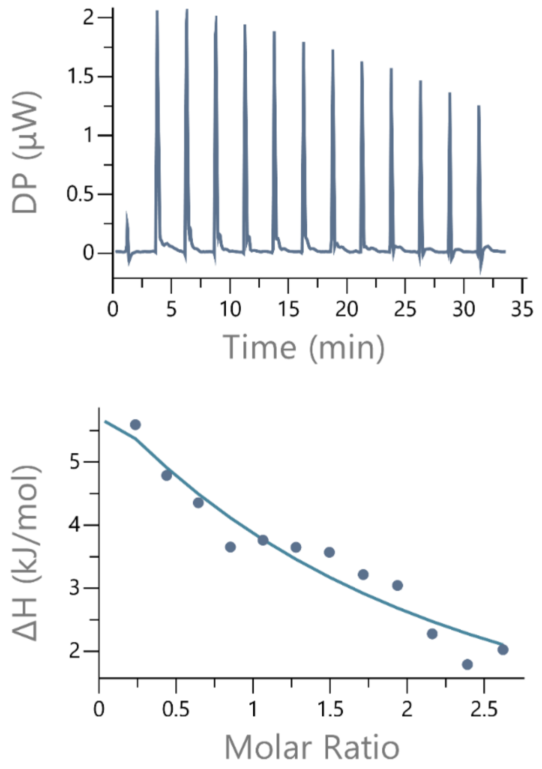

Figure S4. ITC data for a titration of 696 μM HSA into AP solution (50 μM) in phosphate buffer 10 mM, pH = 7.4. *Top:* Raw data; *bottom:* Integrated heat effects per injection as a function of molar ratio (fitted to a one-site model).

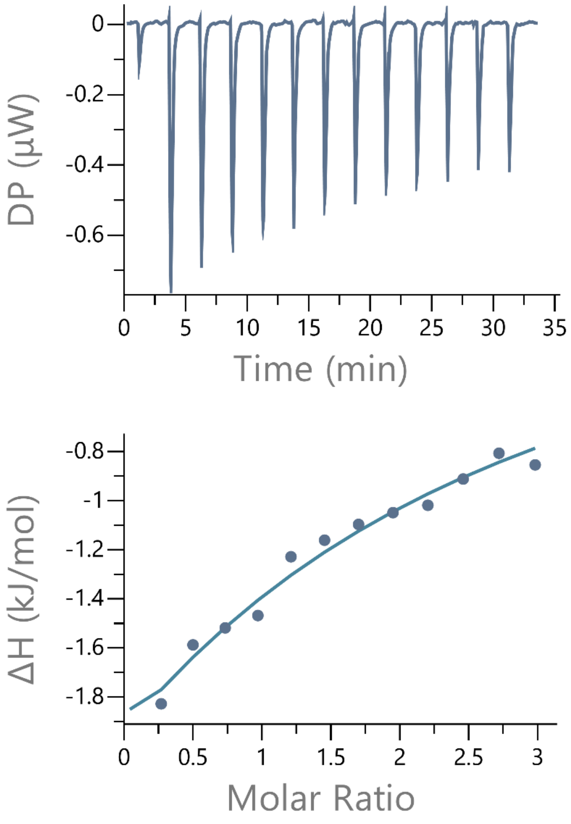

Figure S5. ITC data for a titration of 1.58 mM BE maleate into HSA solution (100 μM) in phosphate buffer 10 mM, pH = 7.4. *Top:* Raw data; *bottom:* Integrated heat effects per injection as a function of molar ratio (fitted to a one-site model).
